# Enhancing Time Series Anomaly Detection: A Knowledge Distillation Approach with Image Transformation

**DOI:** 10.3390/s24248169

**Published:** 2024-12-21

**Authors:** Haiwoong Park, Hyeryung Jang

**Affiliations:** Division of Computer Science & Artificial Intelligence, Dongguk University, Seoul 04620, Republic of Korea; yuti97@dgu.ac.kr

**Keywords:** anomaly detection, time series, imaging time series, knowledge distillation, sensor operation data

## Abstract

Anomaly detection is critical in safety-sensitive fields, but faces challenges from scarce abnormal data and costly expert labeling. Time series anomaly detection is relatively challenging due to its reliance on sequential data, which imposes high computational and memory costs. In particular, it is often composed of real-time collected data that tends to be noisy, making preprocessing an essential step. In contrast, image anomaly detection has leveraged advancements in technologies for analyzing spatial patterns and visual features, achieving high accuracy and promoting research aimed at improving efficiency. We propose a novel framework that bridges image anomaly detection with time series data. Using Gramian Angular Field (GAF) transformations, we convert time series into images and apply state-of-the-art techniques, Reverse Distillation (RD) and EfficientAD (EAD), for efficient and accurate anomaly detection. Tailored preprocessing and transformations further enhance performance and interoperability. When evaluated on the multivariate time series anomaly detection dataset Secure Water Treatment (SWaT) and the univariate datasets University of California, Riverside (UCR) and Numenta Anomaly Benchmark (NAB), our approach demonstrated high recall overall and achieved approximately 99% F1 scores on some univariate datasets, proving its effectiveness as a novel solution for time series anomaly detection.

## 1. Introduction

Anomaly detection involves identifying abnormal data that deviates from standard patterns and plays a critical role in ensuring security and stability in various applications [1]. It is widely adopted in high-stakes fields such as finance, healthcare, and manufacturing, where safety and risk management are priorities. For example, detecting unusual transaction patterns helps prevent fraud in finance, while identifying abnormal patient vitals can enable early diagnosis in healthcare. As a result, anomaly detection remains a research topic of substantial practical value across multiple domains. Despite its importance, anomaly detection faces several inherent challenges. First, the scarcity of anomalous data poses a significant hurdle for training models. Anomalies are rare and unpredictable in real-world settings, making it difficult to collect sufficient data for effective learning. Second, the process of labeling collected data requires specialized knowledge, making it both time-consuming and costly. Lastly, anomalies can appear in diverse and unpredictable patterns, making it nearly impossible to define all abnormal patterns in advance. These challenges highlight the limitations of supervised approaches that rely heavily on labeled examples of anomalies.

To address these challenges, recent research on anomaly detection has increasingly focused on unsupervised learning methods. These approaches train models exclusively on normal data to learn its patterns and classify instances that deviate from these patterns as anomaly during testing. Initially, unsupervised learning methods showed lower detection rates compared to supervised and semi-supervised approaches. However, with advancements in deep learning, applying techniques such as contrastive learning, representation learning, and generative models has led to remarkable detection performance across various domains. Additionally, unsupervised learning approaches often need to process large amounts of data. Techniques like coreset subsampling and knowledge distillation [2] enable efficient memory management, aiding in handling large datasets and enhancing detection performance. These methods enable robust and effective anomaly detection across various applications using only normal data, without requiring labeled anomalies.

With advancements in unsupervised anomaly detection, time series anomaly detection is growing in importance as applications utilizing time series data continue to expand [3]. Time series data, characterized by sequential observations over time, are common in domains such as real-time sensor monitoring, traffic analysis, industrial operations, climate science, and urban planning. Because time series data exhibit temporal dependencies, in which past observations influence current values, specialized approaches that consider both short and long sequences are required for tasks such as forecasting and decision-making. Additionally, time series data accumulate over time, leading to large datasets that often contain noise and missing values, necessitating preprocessing. These factors result in high computational and memory costs for time series anomaly detection and increase the difficulty of detection.

On the other hand, image anomaly detection has achieved high detection performance and efficiency with advancements in image feature learning and modeling techniques. Leveraging generative models and pre-trained models, it has become possible to achieve high-performance anomaly detection by identifying differences that occur in abnormal images. Furthermore, dividing images into patches allows for more precise and detailed anomaly detection. Recent studies have applied techniques such as model lightweight, transfer learning, and knowledge distillation, enabling high efficiency in terms of time and memory while maintaining strong performance.

In this study, we propose a framework that applies image anomaly detection techniques to time series data to achieve high anomaly detection accuracy and computational efficiency. Previous studies have explored the application of time series data to image anomaly detection. For example, T2IAE [4] and TSI-GAN [5] transform time series data into images and employ generative models for anomaly detection. However, these methods rely on relatively outdated models, such as GANs or adversarial autoencoders, in image anomaly detection. ITF-TAD [6] transforms time series data into images using wavelet transformation and applies PatchCore [7], a state-of-the-art method, to image anomaly detection. However, the PatchCore model in ITF-TAD uses feature extractors pre-trained on image datasets, which may not be optimal for images transformed from time series data. We aim to transform time series data into images and apply state-of-the-art image anomaly detection methods to achieve superior performance and efficiency. This process enables the effective representation of normal data patterns from time series as images, addressing the common challenge of insufficient abnormal data in anomaly detection and improving anomaly detection performance. Then, the transformed images are applied to knowledge-distillation-based image anomaly detection techniques to generate anomaly maps. These maps are then used to perform efficient and accurate time series anomaly detection. Our framework modularizes each step, considering the unique characteristics of time series data, and enables the adaptation of methods according to the characteristics of the target dataset to enhance anomaly detection performance. The main contributions of this study are as follows:

- **Generalization of normal patterns through processing:** Normal time series data are segmented based on repetitive patterns and transformed into images while preserving time series characteristics. Although normal patterns in time series data can be unstable due to noise and other factors, segmenting them by cycle and generalizing them into image representations enables more robust detection against such variability. This approach facilitates the formation of normal patterns in unsupervised anomaly detection, leading to improved detection performance.
- **Anomaly-map-based time series anomaly detection:** Using a knowledge-distillation-based anomaly detection model, an anomaly map is derived from image anomaly detection results. The transformed image and the derived anomaly map, which preserve the temporal dependency and time series characteristics, allow for an intuitive understanding of where anomalies occur. Leveraging this map and the preserved time series structure, we propose a more accurate and efficient anomaly detection algorithm for time series data.
- **Experimental validation:** extensive experiments on various real-world datasets validate the effectiveness of the proposed framework and demonstrate significant performance gains over existing methods.

## 2. Related Works

### 2.1. Anomaly Detection of Time Series Data

Time series anomaly detection is a challenging task due to the various characteristics of time series data. One of these characteristics is that past points influence the present, making the order significant and making it challenging to detect anomalies based solely on individual points. Therefore, it is essential to consider the correlations across different time points at the sequence level. Additionally, the characteristics and patterns of time series data can differ across datasets, which can result in considerable variations in performance, even when using the same detection model. For these reasons, time series anomaly detection often shows low accuracy, and developing a generalized model applicable to a wide range of time series datasets is also difficult. To address these challenges, various approaches have been explored for time series anomaly detection, including self-supervised learning, Transformers [8], graph neural networks (GNNs) [9], contrastive learning, and diffusion models [10]. Self-supervised learning [11] and contrastive learning [12] effectively capture patterns and features in normal data, and can be applied to diverse time series datasets, making them highly effective for time series anomaly detection with excellent performance. Transformer-based methods [13,14] are capable of handling long-range dependencies due to the self-attention mechanism, enabling them to effectively learn long-term patterns in time series data. They can also consider relationships between variables. Both of these capabilities contribute to the strong performance in multivariate time series anomaly detection. The traditional transformer had limitations in terms of efficiency, handling temporal dependencies, and real-time anomaly detection. However, recent research has been continuously improving the attention structure and sequence modeling, overcoming these limitations and achieving high performance. Similarly, GNNs are well-suited for capturing complex relationships between variables, making GNN-based methods [15] effective in multivariate time series anomaly detection. However, as the size of the graph increases, the computational cost grows significantly, making it inefficient for large-scale time series data, and it has the limitation of being dependent on the design of the graph structure. Diffusion models learn the distribution of normal time series data and detect anomalies by utilizing reconstruction errors during the restoration process. This allows diffusion-based methods [16] to effectively learn complex patterns in time series data, enhancing their generalization capabilities and reducing the impact of noise. As a result, they achieve a high anomaly detection performance, and can be applied to a wide range of time series datasets. However, due to the high computational complexity, it may be unsuitable for real-time anomaly detection, and since it does not directly capture the temporal dependencies of time series data, it struggles to capture relationships between time points, compared to other models. In this way, various techniques aim to solve the problems in traditional time series anomaly detection by taking into account the characteristics of time series data and enhancing performance. This study was conducted with the same goal.

### 2.2. Image Transformation of Time Series Data

Transforming time series data into images provides a way to apply image anomaly detection techniques to time series data. The purpose of these transformation techniques is to preserve the key temporal features and patterns inherent in time series data while minimizing information loss. Methods like the Markov Transition Field (MTF) [17], Recurrence Plot (RP) [18], and Gramian Angular Field (GAF) [17] are widely used for this purpose. MTF discretizes time series data into states and computes transition probabilities between them, providing a visual representation of temporal transitions. RP calculates the similarity between time points in a time series to construct a recurrence matrix, offering insights into repetitive patterns within the data. GAF encodes time series into polar coordinates, capturing angular information to preserve temporal dependencies and the overall structure of the data. This method has been particularly effective in bridging time series and image anomaly detection. As a result, by selecting the appropriate transformation method based on the characteristics of the dataset, the overall anomaly detection performance can be improved.

### 2.3. Anomaly Detection of Image Data

Image anomaly detection has made significant progress, driven by advancements in feature learning and modeling techniques. In particular, by leveraging generative models, significant progress has been made in overcoming the difficulties of learning complex patterns in image anomaly detection, and it has become possible to interpret anomaly detection results through anomaly maps. Additionally, research on model optimization for efficiency, domain generalization to ensure functionality across various domains, and multimodal approaches continue to be actively explored. Studies such as GANomaly [19] and AnoGAN [20], which utilize Generative Adversarial Networks (GANs), learn the distribution of normal data and effectively detect anomalies by comparing the reconstructed image with the real image. While these methods, using advanced GAN models, can be flexibly applied to various domains, they have limitations, such as unstable training and lower detection performance on complex data. As a result, research continues to explore the integration of GANs with other techniques to overcome these limitations. Additionally, studies like CSFLOW [21] and FastFlow [22], which utilize Normalizing flow, detect anomalies by learning the data distribution and estimating the probability that the data belongs to the normal distribution. This approach has the advantage of providing clear and numerically interpretable anomaly detection criteria, and after training, it offers fast anomaly detection speeds, making it suitable for real-time anomaly detection. However, it has drawbacks, such as complex network design, high training costs, and sensitivity to noise. To address these issues, efforts are being made to improve data preprocessing and architecture design for efficiency. Diffusion-based methods [16] learn the accurate distribution of data, achieving high anomaly detection performance and ease of application across various domains. However, they come with high computational costs and complex training. To address this, model compression and progressive training [23] have been applied, improving both performance and efficiency. Methods using patches, such as SPADE [24] and PaDiM [25], divide images into patches and detect anomalies by comparing features across the patches using pre-trained models on large datasets. This approach makes it easier to detect local anomalies and identify the location of anomalies. However, it is sensitive to patch size, and can be computationally expensive. PatchCore [7] improves efficiency by using a coreset subsampling technique that only stores important features, achieving excellent performance and optimization. ReConPatch [26] employs contrastive learning to learn patch-level representations and calculate the similarity between patches, reducing computational complexity while achieving high anomaly detection accuracy. Additionally, GLASS [27] introduces a different image anomaly detection approach by generating artificial anomalous data and amplifying the differences between normal and anomalous data to effectively learn the patterns of normal data and achieve high anomaly detection performance. In this way, image anomaly detection has demonstrated high accuracy, and recent research is actively focusing on achieving efficiency as well.

## 3. Preliminaries

In this section, we describe two knowledge-distillation-based image anomaly detection models that we used to derive the anomaly map.

### 3.1. Reverse Distillation

**Figure 1:**
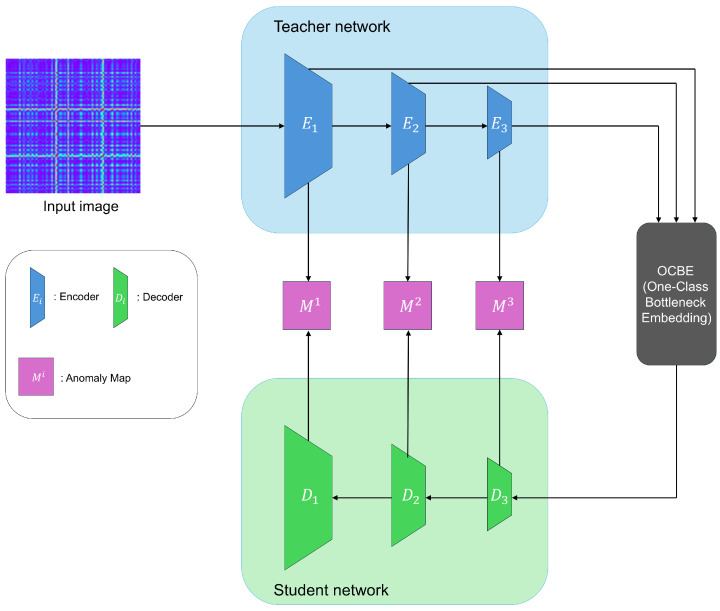
Overview of reverse distillation.

Reverse Distillation (RD) [28] is designed to ensure that the teacher and student models do not share the same architecture and have different data flows. This resolves the problem of the student model excessively mimicking the teacher model in traditional knowledge-distillation-based anomaly detection, which often leads to failures in detecting anomalies. Figure 1 illustrates the overall structure of Reverse Distillation.

Reverse Distillation employs a reversed architecture, using a pre-trained encoder as the teacher model and a trainable decoder as the student model. This design prevents the student model from directly mimicking the parameters of the teacher model. Additionally, it introduces a module called the One-Class Bottleneck Embedding Module (OCBE), which compresses the features extracted by the Teacher model before passing them to the Student model. During this process, the Student model filters out unnecessary information, such as anomalies and noise, and learns only the information relevant to normal data. This ensures that the student model does not mimic the teacher model for abnormal data. The training process uses only normal data and employs a loss function to minimize the difference between the features encoded by the Teacher model and those decoded by the Student model. The feature differences are calculated at each layer using pixel-level cosine similarity, and these differences are then used to generate anomaly maps for each layer.

(1) $$M^{k}\left(h,w\right)=1-\frac{{\left(f_{E}^{k}\left(h,w\right)\right)}^{T}\cdot f_{D}^{k}\left(h,w\right)}{∥f_{E}^{k}\left(h,w\right)∥∥f_{D}^{k}\left(h,w\right)∥},$$

where $f_{E}^{k}\left(h,w\right)$ and $f_{D}^{k}\left(h,w\right)$ represent the feature vectors at spatial location (h,w), ∥·∥ denotes the Euclidean norm, *E* corresponds to the encoder of the teacher model, *D* represents the decoder of the student model, *k* denotes the layer index, and h,w specify the spatial pixel locations.

(2) $$L_{\mathrm{KD}}=\sum_{k=1}^{K}\left\{\frac{1}{H_{k}W_{k}}\sum_{h=1}^{H_{k}}\sum_{w=1}^{W_{k}}M^{k}\left(h,w\right)\right\},$$

where $L_{\mathrm{KD}}$ represents the knowledge distillation loss, where *K* is the total number of layers considered, and $H_{k}$ and $W_{k}$, respectively, denote the height and width of the anomaly map at the *k*-th layer.

Since the anomaly maps generated by each layer have different resolutions, they are up-sampled and then combined to produce the final anomaly map for the input image. This approach overcomes existing limitations and enables accurate anomaly detection.

### 3.2. EfficientAD

**Figure 2:**
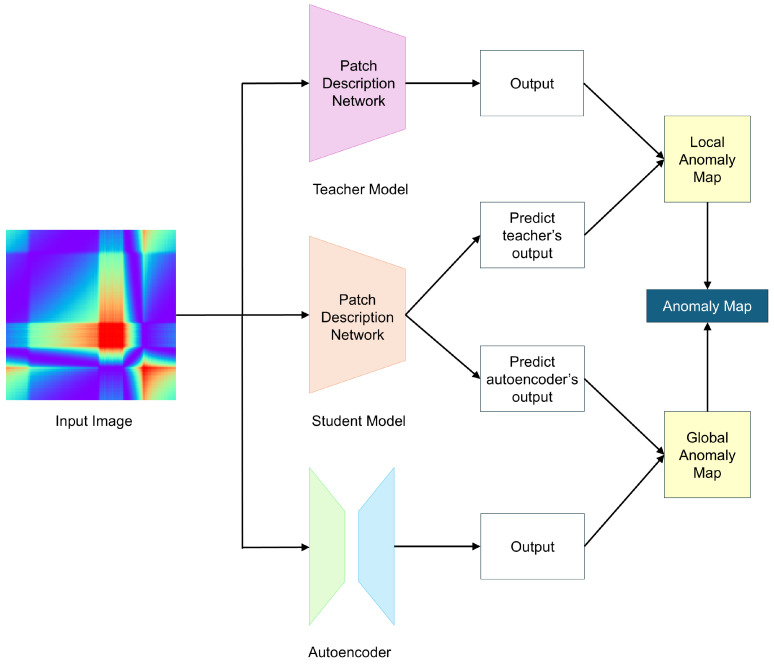
Overview of EfficientAD.

EfficientAD (EAD) [29] utilizes a lightweight feature extractor called the Patch Description Network (PDN), which efficiently represents each 33 × 33 patch as a single feature vector, enabling efficient anomaly detection. Similar to other methods, EAD uses only normal data during training and detects anomalies based on the differences between the outputs of the teacher and student models. Figure 2 illustrates the overview of the EAD architecture.

While using a lightweight feature extractor can improve efficiency, it may lead to lower anomaly detection performance. To address this, EAD introduces a hard feature loss to enhance detection performance. The hard feature loss, $L_{\mathrm{hard}}$, is designed to compute the loss only for areas where the student model fails to properly mimic the teacher model during training. $L_{\mathrm{hard}}$ is defined as

(3) $$L_{\mathrm{hard}}=\frac{1}{|D_{\mathrm{hard}}|}\sum_{\left(c,w,h\right)\in D_{\mathrm{hard}}}D_{c,w,h},$$

where $D_{c,w,h}$ represents the distance between the teacher model’s output and the student model’s output at the *c*-th channel and position (w,h), with *I* denoting the input image, quantified as

(4) $$D_{c,w,h}={\left(T{\left(I\right)}_{c,w,h}-S{\left(I\right)}_{c,w,h}\right)}^{2}$$

and $D_{\mathrm{hard}}$ is the set of values in *D* that are in the top $p_{\mathrm{hard}}$ percentile. Additionally, EAD introduces a loss penalty term called $L_{\mathrm{penalty}}$. Features are extracted from images randomly sampled from a dataset not used during training, and the average of these features is used as $L_{\mathrm{penalty}}$. This ensures that data not seen during training yield high loss values, preventing the student model from effectively mimicking the teacher model for images that are either abnormal or unseen during training.

(5) $$L_{\mathrm{penalty}}=\frac{1}{CWH}\sum_{c}{∥S{\left(P\right)}_{c}∥}_{F}^{2},$$

where *C*, *W*, and *H* are the number of channels, width, and height of the output, and *P* represents a randomly sampled image from a dataset not used during training. Consequently, the loss $L_{\mathrm{ST}}$, which represents the difference between the student model and the teacher model in EAD, is defined as

(6) $$L_{\mathrm{ST}}=L_{\mathrm{hard}}+L_{\mathrm{penalty}}.$$

EAD also utilizes an autoencoder to detect logical anomalies. Logical anomalies involve contextual issues in an image, such as objects being in unexpected positions or having abnormal sizes. It refers to an anomaly that can only be identified by analyzing the image as a whole. To detect this, an autoencoder is trained using distance loss, $L_{\mathrm{AE}}$, with the teacher’s output as the target. $L_{\mathrm{AE}}$ is defined as

(7) $$L_{\mathrm{AE}}=\frac{1}{CWH}\sum_{c}{∥T{\left(I\right)}_{c}-A{\left(I\right)}_{c}∥}_{F}^{2},$$

where $A{\left(I\right)}_{c}$ denotes the output of the autoencoder for the *c*-th channel.

If a logical anomaly is present in the image, it can be detected due to the failure in reconstruction. However, autoencoders often have difficulty capturing fine-grained patterns, leading to reconstruction failures even for normal images, which can cause false positives. To prevent this, the student model’s output is doubled, enabling it to predict not only the teacher’s output, but also the autoencoder’s output. Additionally, the difference between the student model’s output and the autoencoder’s output is used as an additional loss term, $L_{\mathrm{STAE}}$, to reduce false positives and stabilize the training process. $L_{\mathrm{STAE}}$ is defined as

(8) $$L_{\mathrm{STAE}}=\frac{1}{CWH}\sum_{c}{∥A{\left(I\right)}_{c}-S^{\prime }{\left(I\right)}_{c}∥}_{F}^{2},$$

where $S^{\prime }{\left(I\right)}_{c}$ denotes the student model’s output that predicts the autoencoder’s output for the *c*-th channel, and $A{\left(I\right)}_{c}$ represents the autoencoder’s output for the same channel. Finally, the total loss for EAD is defined as

(9) $$L_{\mathrm{total}}=L_{\mathrm{ST}}+L_{\mathrm{AE}}+L_{\mathrm{STAE}}.$$

After training, the student model, utilizing the PDN, has a short dependency range, while the autoencoder has a long dependency range. This difference in long-range dependencies makes it possible to generate a global anomaly map for detecting logical anomalies. Additionally, since both the student model and teacher model have short dependency ranges, they can be used to generate a local anomaly map. EAD combines the global anomaly map and the local anomaly map to produce the final anomaly map.

## 4. Methods

**Figure 3:**
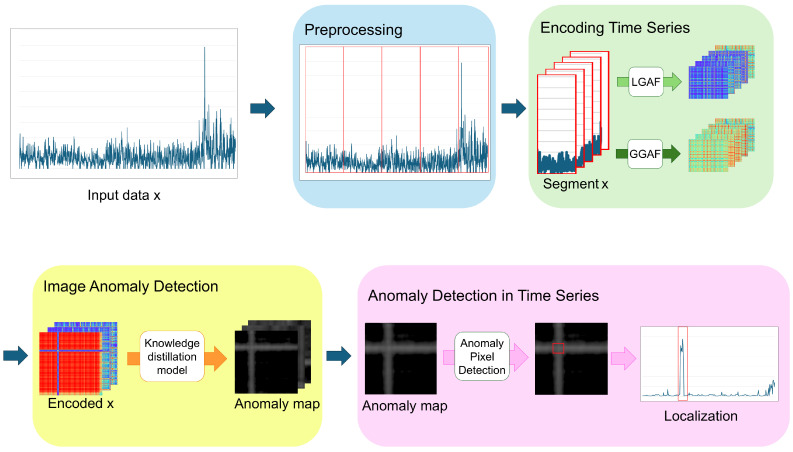
The overall structure of the proposed framework.

The overall structure of the framework proposed in this study is illustrated in Figure 3, and each component is described in detail in the following subsections. Section 4.1 discusses the pre-processing step, where time series data are transformed and segmented according to its characteristics before being converted into images. In Section 4.2, we explain the method for converting the pre-processed time series data into images. Section 4.3 describes the process of generating anomaly maps from the transformed images using knowledge-distillation-based image anomaly detection models. Lastly, in Section 4.4, we explain the method for performing time series anomaly detection using the generated anomaly maps.

### 4.1. Data Pre-Processing

**Figure 4:**
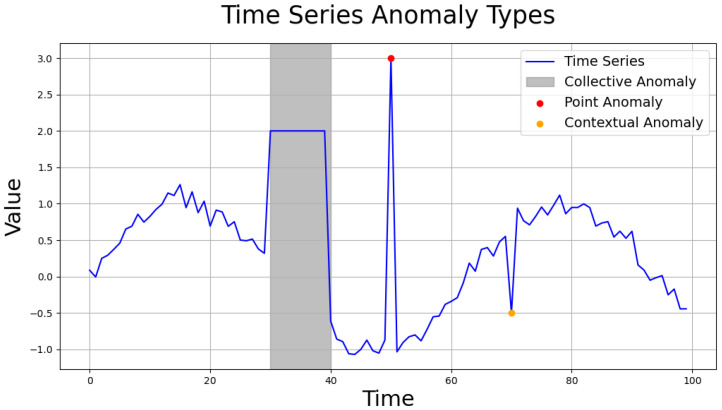
Anomaly types of time series data.

Anomalies in time series data can manifest in various forms depending on the dataset’s characteristics [30]. Typically, these anomalies are categorized into three types (see Figure 4 for examples):

- Point anomaly: abnormal data values at specific time points.
- Contextual anomaly: data with individually normal values that appear abnormal in a temporal context.
- Collective anomaly: a group of data points that individually appear normal but together form an anomalous pattern.

Contextual anomalies and collective anomalies can be challenging to detect because individual values may appear as normal data. Additionally, real-world time series data are often vast, making labeling difficult and containing noise and missing values, which complicates anomaly detection. Despite these challenges, detecting all anomalies is crucial as they can lead to substantial losses. To address these limitations and improve anomaly detection, we explain data transformation methods that can enhance detection performance by considering the characteristics of the data during the pre-processing stage. We also describe a method for dividing the time series into subsequences based on cycle to generalize normal patterns. This segmentation plays a significant role in generalizing unstable normal patterns, which are affected by noise and other factors.

#### 4.1.1. Data Transformation

Let $x_{t}$ denote the observed value at time *t* within the time series $x=\{x_{1},x_{2},\ldots ,x_{T}\}$, where *T* represents the total number of observations. Abnormal data are unpredictable, and may exhibit either large or small deviations from normal patterns. Anomalies with large deviations can mask anomalies with relatively smaller deviations, reducing the anomaly detection performance. Conversely, very small deviations can be difficult to distinguish from normal data, making them undetectable. Therefore, transformations that amplify or reduce deviations between data points, considering the characteristics of the data, can be beneficial for anomaly detection. We provide a brief explanation of three representative transformation methods.

1. Min-Max Normalization scales data to a range of [−1,1], enhancing small differences between values for improved anomaly detection. However, it may distort distributions in the presence of significant discrepancies. The transformation process is expressed as follows:
  (10) $$x_{t,\mathrm{minmax}}=\frac{x_{t}-\min\left(x\right)}{\max(x)-\min(x)}\times 2-1,$$
  where min(x),max(x) are the minimum and maximum values in the time series x, respectively.
2. Z-Score Normalization adjusts the data to have a mean of zero and scales it by the standard deviation, making it easier to identify anomalies that differ significantly from the average. This method may underperform for non-normal distributions or datasets heavily influenced by anomalies. The transformation process is defined as follows:
  (11) $$x_{t,z\text{-}\mathrm{score}}=\frac{x_{t}-\mu }{\sigma },$$
  where μ is the mean of the times series x and σ is the standard deviation of the time series x.
3. Log Transformation reduces the impact of extreme values and stabilizes data distribution. It is particularly useful for wide value ranges, but may distort cyclic patterns. The log transformation process is represented by the following equation:
  (12) $$x_{t,\log}=\log\left(x_{t}+1\right).$$

In addition to the three methods mentioned above, other techniques, such as Fourier transform and wavelet transform, can be applied based on the characteristics of the dataset. Fourier transform converts time series data into the frequency domain, facilitating the detection of cyclic patterns, while wavelet transform provides both time and frequency information, enabling effective analysis of various patterns within the data. Applying these transformations in alignment with the dataset characteristics can further improve anomaly detection performance.

#### 4.1.2. Time Series Segmentation

Time series data exhibit temporal dependencies, where past values influence current ones. However, analyzing the entire sequence increases model complexity and reduces efficiency. Dividing the data into subsequences for analysis can address these issues to some extent. When segmenting the data, the criteria for division are quite important. Since the temporal correlations between time points in the data must be considered, the subsequences should not be too short. Conversely, if the subsequences are too long, the aforementioned issues may arise. Additionally, if segmentation is performed without considering the repetitive patterns inherent in the time series data, it can make pattern analysis more challenging. To preserve temporal dependencies effectively and make the data suitable for unsupervised anomaly detection, we segmented the data based on the intervals where repetitive patterns appear. These intervals are called cycles. Since there can be multiple types of patterns, and the intervals are not always consistent, determining cycle by considering all patterns is difficult and ambiguous. Such cycles can be defined using intervals between successive extrema or mean values, and can also be determined using domain knowledge, time series decomposition, and frequency analysis.

**Figure 5:**
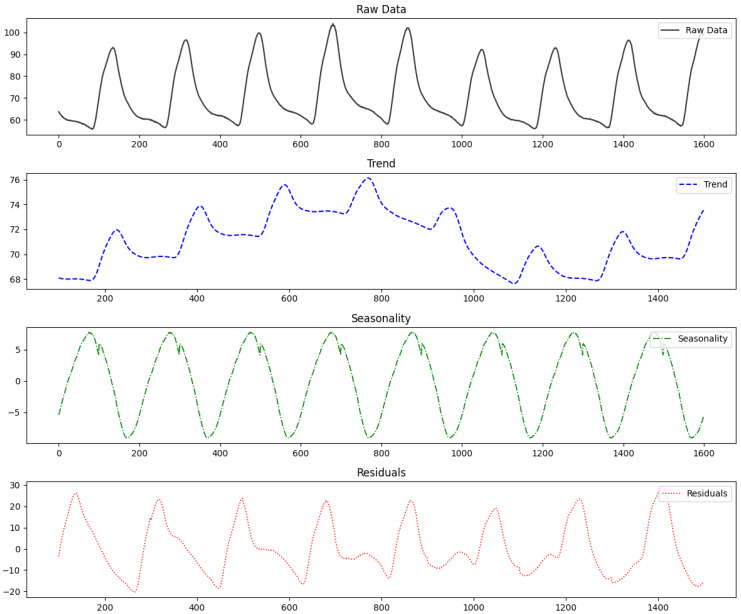
Time series decomposition results.

Figure 5 shows the result of decomposing time series data into trends, seasonality, and residuals. Adjusting the intervals of the cycle using decomposition results, domain knowledge, and frequency analysis helps in identifying the appropriate cycle. This allows the time series data to be segmented by pattern, significantly improving the learning level for normal patterns. Additionally, it enables the discovery of patterns that are difficult to intuitively identify in time series data. This approach of segmenting time series data by cycles holds significant value in anomaly detection. However, for non-cyclic data, the sequence is divided into fixed-length segments. In this case, it becomes challenging to account for the patterns in the time series data, which can hinder pattern analysis and result in lower detection performance. The segmented intervals are represented as follows:

(13) $${\mathrm{Segment}}_{i}=\left\{x_{t}|t_{i}\leq t<t_{i+1}\right\},$$

where *t* represents the time points, $t_{i}$ and $t_{i+1}$ denote the boundaries of the interval for the defined cycle.

Even when cycles exist, it can be difficult to establish criteria for cycle segmentation if there are various types of repetitive patterns with significant detailed differences, even though they appear similar in the overall context. In such cases, a domain expert or user manually defines the intervals for segmentation. This approach requires a process similar to labeling, as segmentation must be performed for each cycle, which can be inefficient. However, if insightful segmentation is achieved, it can result in high detection rates, even for complex data. The segmented intervals are represented as follows:

(14) $${\mathrm{Segment}}_{i}=\left\{x_{t}|a_{i}\leq t\leq b_{i}\right\},$$

where $a_{i},b_{i}$ are the starting and end points of the segment defined by the user, respectively.

As a result, the approach of segmenting cycles aims to divide time series data into meaningful patterns, enabling each subsequence to effectively represent the time series patterns and enhance anomaly detection performance.

### 4.2. Encoding Time Series

In this study, we employ Gramian Angular Field (GAF) [17] transformation to convert time series data into images while preserving temporal dependencies. Two versions are utilized:

- **Local GAF (LGAF)**: focuses on local extrema for normalization, emphasizing subtle changes.
- **Global GAF (GGAF)**: normalizes using global extrema, providing robustness to noise and better generalization.

The conversion process of LGAF involves first normalizing the time series data to a range of [−1,1]. The normalized values are then transformed into angles in a polar coordinate system.

Given a normalized time series $\tilde{x}=\left\{{\tilde{x}}_{1},{\tilde{x}}_{2},\ldots ,{\tilde{x}}_{T}\right\}$, each element ${\tilde{x}}_{i}$ is transformed into an angle $\phi_{i}$ as

$$\phi_{i}=\arccos\left({\tilde{x}}_{i}\right),\;\mathrm{for}\;i\;=\;1,2,\ldots ,n\;(n\;=\;\mathrm{subsequence}\;\mathrm{length}).$$

Then, a symmetric matrix is constructed using either sum or difference of angles between two time points. Two types of matrices can be generated: Gramian Angular Summation Field (GASF), which computes the cosine of the angle sums, and Gramian Angular Difference Field (GADF), which computes the sine of the angle differences. GASF emphasizes the correlation between time points in a time series and preserves overall patterns, while GADF captures changes in the time series, providing a clearer representation of dynamic variations. In this study, we used only GASF, as the data consists of repetitive signal values, making it more suitable for analyzing cyclic patterns in time series data. As a result, the local GAF (LGAF) is obtained as follows:

(15) $$\mathrm{LGAF}=\left[\begin{array}{cccc} \cos(\phi_{1}+\phi_{1}) & \cos(\phi_{1}+\phi_{2}) & \cdots & \cos(\phi_{1}+\phi_{n})\\ \cos(\phi_{2}+\phi_{1}) & \cos(\phi_{2}+\phi_{2}) & \cdots & \cos(\phi_{2}+\phi_{n})\\ \vdots & \vdots & \ddots & \vdots \\ \cos(\phi_{n}+\phi_{1}) & \cos(\phi_{n}+\phi_{2}) & \cdots & \cos(\phi_{n}+\phi_{n}) \end{array}\right]$$

However, images generated through the LGAF transformation cannot account for the information of the entire sequence. To address this limitation, we propose a modified image transformation method called Global GAF (GGAF), which incorporates the information of the entire sequence. GGAF normalizes the time series data to the range of [−1,1] for conversion into a polar coordinate system, but it includes the global maximum and minimum values of the entire sequence during the normalization process. By considering the full range of the sequence values, GGAF reduces the influence of local extrema and transforms the time series into images that reflect the overall sequence more comprehensively. In GGAF, the time series data are transformed into an angle as

$$\phi_{i}=\arccos\left({\tilde{x}}_{i}\right),\;\mathrm{for}\;i=1,2,\ldots ,N\;(N\;=\;\mathrm{total}\;\mathrm{sequence}\;\mathrm{length}).$$

**Figure 6:**
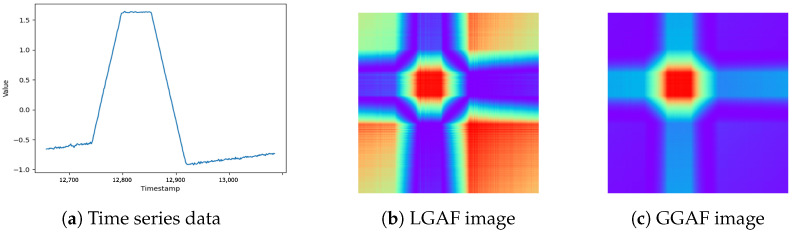
Comparison of time series data and GAF representations.

LGAF is better at capturing subtle changes, but can be heavily influenced by local extrema. On the other hand, GGAF considers the entire range of time series data, making it more robust to noise and capable of representing the data in a more generalized form. However, GGAF becomes less effective at detecting subtle changes, and may result in decreased performance if the overall range of the data are too wide, leading to model insensitivity and overfitting. Both methods aim to effectively represent the normal patterns of time series data. By analyzing the characteristics of the data before transformation and applying the appropriate method, detection performance can be improved. In Figure 6, we provide examples of time series data and transformed images obtained using LGAF and GGAF.

### 4.3. Image Anomaly Detection via Knowledge Distillation

In the previous step, the time series data were encoded into images while preserving its temporal dependencies and correlations. Using the images transformed by the Gramian Angular Field (GAF), this step applies a knowledge-distillation-based image anomaly detection model.

Knowledge Distillation (KD) [2] is a widely used technique for making deep learning models more efficient and lightweight. This approach leverages the relationship between a teacher model and a student model during training. The teacher model is typically a large, pre-trained network trained on a large scale dataset, demonstrating high performance. On the other hand, the student model is designed as a much smaller network, which is trained to mimic the outputs of the teacher model. By transferring knowledge from the teacher model to the student model, the student model can achieve results close to the teacher model’s performance while requiring fewer computational resources and less memory. In other words, knowledge distillation transfers the complex learning and inference abilities of the large teacher model to the smaller student model, allowing the student model to closely replicate the performance of the teacher model.

This knowledge distillation technique has also been applied to anomaly detection. In anomaly detection, the basic approach to leveraging knowledge distillation [31] is to train the student model to mimic the teacher model only on normal data. The student model learns to replicate the teacher model’s outputs well for normal data, but fails to do so for abnormal data that it has not encountered during training. This leads to a discrepancy between the outputs of the teacher and student models for abnormal data, which is then used to detect anomalies. In image anomaly detection, this discrepancy is used to create an anomaly map in the form of a heatmap. However, this approach can sometimes fail when the student model generalizes the teacher model’s outputs too well, even for abnormal data, leading to a failure in detection. Research on anomaly detection using knowledge distillation has aimed to address this issue. Among the proposed methods, Reverse Distillation (RD) [28] prevents the student model from perfectly mimicking the teacher model by altering their structures, and EfficientAD (EAD) [29] ensures that the student model learns only the parts where it fails to replicate the teacher model’s outputs, effectively overcoming this limitation. RD generates anomaly maps at multiple resolutions, upscales them into a final anomaly map, and utilizes it for precise anomaly detection. EAD uses a lightweight feature extractor, Patch Description Network (PDN), for efficiency and integrates an autoencoder to detect logical anomalies as well. In time series data, a logical anomaly is typically considered a type of contextual anomaly, as the data may appear normal in isolated segments but reveal logical inconsistencies when viewed in the overall context. EAD can detect such logical anomalies by incorporating an autoencoder. It combines the global map, which captures the logical anomaly, with the local map, which identifies localized anomaly, to produce a final anomaly map.

Since we transformed the time series data into images while preserving their temporal characteristics, we believe that applying image anomaly detection methods to these transformed images allows for anomaly detection while considering the temporal characteristics of the data. Therefore, we utilize the anomaly maps generated from the transformed images for time series anomaly detection. To achieve accurate and efficient anomaly detection, we adopt image anomaly detection techniques that generate precise and efficient anomaly maps, leveraging RD and EAD models. By utilizing the anomaly maps derived from these models, we aim to perform highly accurate and efficient time series anomaly detection.

### 4.4. Anomaly Detection in Time Series

This study preprocesses time series data by segmenting it into cyclic patterns. This step divides the time series data into segments based on cycles, allowing for the formation of robust generalized patterns that are less affected by fluctuations. The segmented subsequences are then converted into images using LGAF or GGAF, preserving temporal order and correlations. This enables the application of high-performance image anomaly detection methods while retaining the essential temporal characteristics required for handling time series data. In this study, anomaly maps for the transformed images are generated using a knowledge-distillation-based image anomaly detection model. All these processes aim to enhance anomaly detection performance and adapt time series data to advanced unsupervised image anomaly detection methods. The pseudocode for these processes can be found in Algorithm 1.    
**Algorithm 1:** Anomaly map generation via preprocessing, GAF, and knowledge distillation model
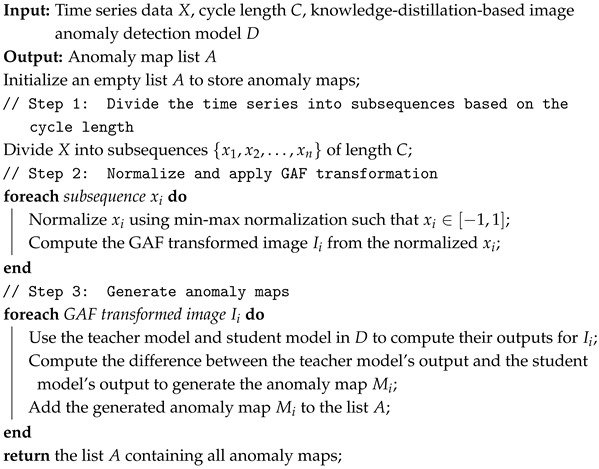


Since the primary goal of this study is time series anomaly detection, the anomaly maps obtained through the previous processes must be utilized for detecting anomalies in time series data. The width and height of a GAF transformed image are identical to the length of the subsequence it represents. In the image, each pixel indicates the relationship between the time points corresponding to its coordinates. For example, in an image *I*, the pixel *I*(32, 48) represents the relationship between the 32nd and 48th time points. This also applies to the anomaly map, where the pixel values represent how abnormal the relationship is between the two time points corresponding to the coordinates. By examining the pixel values in the anomaly map, we can determine how much the relationship between each time point and other time points deviates from the normal pattern. In other words, it reveals how much a specific time point deviates from the overall pattern of the subsequence. An anomaly is a time point that deviates from the normal pattern and is therefore likely to have relationships with other time points in the subsequence that differ significantly from those of normal data. Thus, by analyzing the relationships between each time point and other time points in the anomaly map, it is possible to detect whether a specific time point is an anomaly.

Before applying the previously described anomaly detection rule, this study employs an initial anomaly detection criterion to ensure efficient detection. For cyclic data, the entire sequence is divided into subsequences based on cycles. The length of each divided subsequence represents the cycle length. If a sequence does not exhibit a normal pattern, it will face difficulty in this cycle-based segmentation, resulting in abnormal subsequence lengths. Therefore, if the length of a subsequence deviates significantly from the average subsequence length used during training, it is detected as an anomaly.

If the subsequence length is considered normal, the pixel values in the anomaly map are examined. As detecting anomalies for all pixels is inefficient, the detection begins with the pixels on the main diagonal. In the transformed image, pixels on the main diagonal represent the relationship of a time point with itself. If a time point is an anomaly, it is likely to have relationships with other time points that deviate from the normal pattern, resulting in higher values in the anomaly map. Therefore, anomaly detection is performed only for the time points where the pixel values on the main diagonal exceed the threshold. For the selected time points, the values of all pixels in the same row and column as the main diagonal pixel are summed. This is because the pixels in the same row and column contain information about the relationships between the selected time point and other time points. The values of all these pixels are summed to calculate the Final Anomaly Score (FAS). If the FAS exceeds threshold·number of pixels·*H*, the selected time point is classified as an anomaly. *H* is a hyperparameter designed to relax the anomaly detection criteria. This variable helps detect subtle or continuous anomalies, which might have pixel values relatively close to those of normal patterns. By tuning *H*, the success rate of detecting such cases improves, which enhances overall anomaly detection performance. The pseudocode for this process is presented in Algorithm 2.

The above detection method is applied to each image, producing detection results for each subsequence. These results are then combined to generate the final anomaly detection outcome for the entire sequence. This study adopts and develops methodologies that are both efficient and enhance detection performance at each section, resulting in a robust and efficient time series anomaly detection system.
**Algorithm 2:** Time series anomaly detection using anomaly map
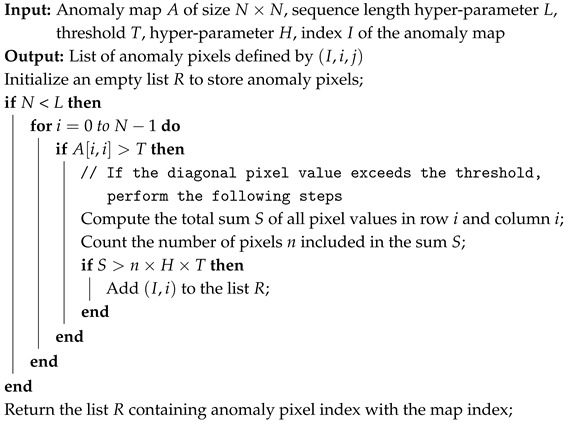


## 5. Experiments

### 5.1. Experimental Setup

**Datasets**. To evaluate performance, we used three publicly available time series datasets. These widely used benchmark datasets were utilized to compare our results with other methods. The datasets are summarized in Table 1.

- Secure Water Treatment dataset (SWaT) [32]: The SWaT dataset consists of data collected from 51 sensors in a real water treatment process. Each sensor measures parameters such as water level, pressure, flow rate, and actuator operation. Only the normal operation data were used for training, while the abnormal operation data were used for testing. Testing was performed individually on representative sensors, and the results from these sensors were combined to determine the overall anomaly detection performance for the SWaT dataset. According to [32], the system requires approximately 5 h for stabilization. Therefore, the first 21,600 data points were removed from the training data, and down-sampling by a factor of 10 was applied to process the data efficiently.
- InternalBleeding dataset from UCR [33]: The University of California, Riverside (UCR) dataset includes time series data collected from various domains, such as industrial systems, environmental sensors, biological signal sensors, and financial data. It is widely used for time series anomaly detection research. The dataset consists of uni-variate time series data. In our experiments, we used the InternalBleeding data, which contains measurements of arterial blood pressure in pigs, excluding synthetic sequences generated with intentionally created anomalies.
- NAB dataset [34,35]: The Numenta Anomaly Benchmark (NAB) dataset comprises real and artificially generated uni-variate time series data collected to evaluate anomaly detection algorithms for real time applications. It includes data such as machine temperature sensor readings, CPU temperatures, New York taxi traffic volumes, and AWS server metrics. For our experiments, we divided the data into subsequences of length 100 and performed anomaly detection. Only normal data points, excluding 233 anomaly containing timestamps, were used for training.

**Metrics**. The results of time series anomaly detection were evaluated by comparing the actual labels with the detected anomalies, defined as follows:

- True Positive (TP): an instance where an actual abnormal point is correctly identified as abnormal.
- False Positive (FP): an instance where a normal point is incorrectly identified as abnormal.
- True Negative (TN): an instance where a normal point is correctly identified as normal.
- False Negative (FN): an instance where an actual abnormal point is incorrectly identified as normal.

Based on these cases, the performance of the time series anomaly detection system in this study was evaluated using various metrics, including Accuracy, Precision, Recall, F1 score, False Positive Rate (FPR), and Area Under the Receiver Operating Characteristic Curve (AUROC). These metrics were designed to assess the anomaly detection performance from different perspectives, particularly for imbalanced datasets with relatively few anomalies. Through this approach, the comprehensive performance of the time series anomaly detection system was evaluated.

**Experimental environment**. In this study, PyTorch [36] versions 1.13.0, 1.9.1, and 2.4.1 were used due to the presence of modules requiring different virtual environments. All experiments were conducted on a server equipped with CUDA 12.1 and four NVIDIA GeForce RTX 3090 GPUs. The EAD model was trained using the Adam optimizer [37] with a learning rate of $1\times 10^{-4}$ and momentum parameters $\left(\beta_{1},\beta_{2}\right)=\left(0.9,0.999\right)$. The RD model was also trained using the Adam optimizer, but with a learning rate of 0.005 and momentum parameters $\left(\beta_{1},\beta_{2}\right)=\left(0.5,0.999\right)$.

### 5.2. Analysis of the Proposed Framework

**Figure 7:**
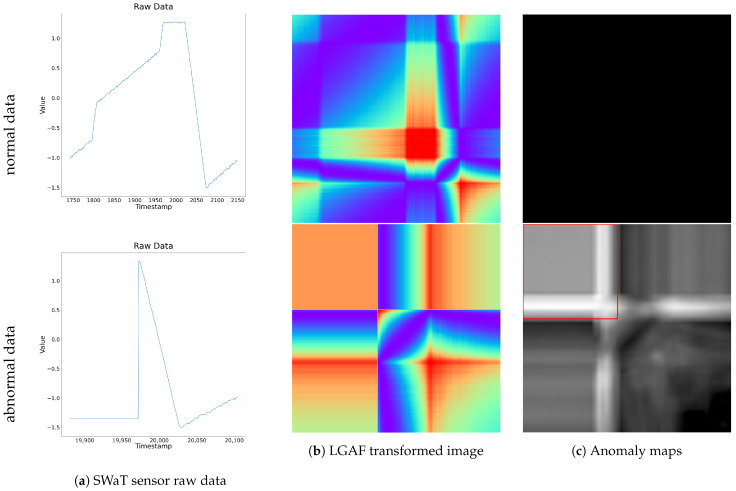
Visualization of SWaT raw data, LGAF transformed images, and anomaly maps for normal and abnormal data segments. The top row corresponds to the normal data segment, and the bottom row represents the abnormal data segment.

Figure 7 illustrates a specific segment of the LIT-301 sensor data from the SWaT dataset. The top row represents normal data, while the bottom row represents abnormal data containing anomalies. From left to right, each column displays the raw time series data, the LGAF transformed image, and the resulting anomaly map. For the normal data, the anomaly map shows an anomaly score of 0 for all pixels, indicating no detected anomalies at any time point. In contrast, the anomaly map for the abnormal data contains multiple pixels along the main diagonal that exceed the threshold, and the FAS value also surpasses the criteria, successfully detecting anomalies. These detected points correspond to time segments in the raw data where abnormal patterns deviate from the normal patterns. This demonstrates the successful anomaly detection process. The detailed results of anomaly detection are presented below.

#### 5.2.1. Module-Wise Analysis

This section evaluates the performance of our proposed system on the dataset, compares the performance of different modules, and provides an interpretation of the results. The experimental results include comparisons of segmentation methods, encoding techniques, and utilized models. For all configurations other than the comparison targets, the settings achieving the highest performance were selected for the experiments.

**Figure 8:**
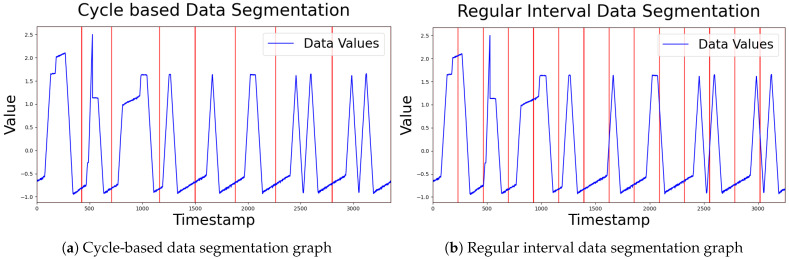
Comparison of data segmentation methods.

Table 2 presents the experimental results comparing time series data segmentation based on cyclic intervals with segmentation using regular intervals. Due to the nature of the proposed method, it is hard to consider the interrelations between different time series segments. However, segmenting data by cyclic intervals enables each subsequence to represent a typical normal pattern, which significantly contributes to improving overall anomaly detection performance even without considering such interrelations. Moreover, this approach preserves temporal relationships within a cycle, such as time dependencies within the data, further enhancing detection accuracy. That said, this method is difficult to apply to non-cyclic data and can degrade performance if an incorrect cycle is selected. Therefore, to effectively apply cyclic segmentation, preliminary processes such as data analysis or decomposition are essential. Figure 8 shows examples of dividing the image by cycle and by regular interval.

Table 3 presents the experimental results comparing different image encoding methods. GGAF has an advantage in maintaining consistency within time series data, reducing the impact of noise, and preserving overall patterns. However, in the SWaT dataset used in this study, the sensor values exhibit oscillations with small variations and are not significantly affected by extreme values. Due to these data characteristics, the experimental results for LGAF and GGAF showed no substantial differences. This is because the stable variability in the data did not provide factors that could clearly distinguish the two methods. Similarly, no significant differences were observed in the UCR dataset.

In contrast, for the NAB dataset, the LGAF method failed to detect one of the three anomalous points, whereas the GGAF method successfully detected all three. This can be attributed to the oscillatory nature of normal data within a specific range, where GGAF’s ability to apply consistent criteria and reduce the influence of minor variations made it more effective at identifying anomaly caused by extreme values.

**Figure 9:**
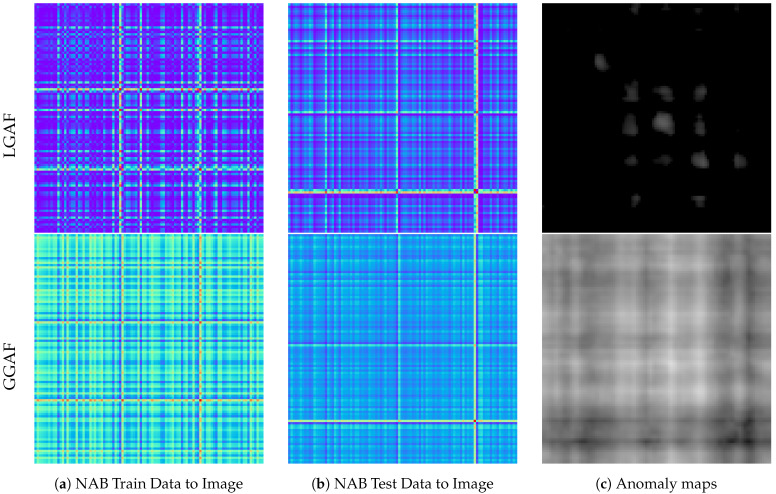
Comparison of LGAF and GGAF on NAB train and test data, including anomaly maps. The top row shows results from LGAF, and the bottom row shows results from GGAF.

Figure 9 illustrates the results of LGAF and GGAF on the NAB dataset. The top row presents the results using LGAF, and the bottom row shows the results using GGAF. Images generated with LGAF present minimal differences between anomalous and normal data, resulting in low anomaly scores on the corresponding anomaly maps. In contrast, images generated with GGAF present more pronounced differences, and the anomaly maps show higher anomaly scores, successfully identifying the anomalies.

As such, GGAF may be more effective for detecting anomalies associated with extreme values or in scenarios where the data exhibits high variability and noise.

Table 4 presents the experimental results comparing different image anomaly detection models. The results focus on univariate data for each model, showing similar performance across the models with no significant differences for the dataset used in this study. As shown in Table 5, RD, despite its simple structure, calculates feature differences across multiple layers. On the other hand, EAD uses the efficient PDN network, resulting in lower memory usage compared to RD.

In terms of training time, there were notable differences between the two models. RD employs a standard Dataloader, leading to relatively shorter training times. In contrast, EAD utilizes an InfiniteDataloader, which allows for infinite repetition of the dataset during training, resulting in longer training times. However, since EAD is not influenced by the number of training samples, it could potentially achieve shorter training times when handling large datasets.

Given that the dataset used in this study primarily consists of simple patterns, the performance gap between EAD and RD was not significant. In conclusion, EAD is more suitable for efficient anomaly detection tasks with low memory requirements, while RD is better suited for detecting anomalies in complex datasets where higher precision is required.

#### 5.2.2. Comparison with Baselines

This section compares the performance of the proposed system on the dataset with various baselines and provides an interpretation of the results. Similarly to the previous section, experiments were conducted using configurations that achieved the highest performance. However, a new criterion for the anomaly detection threshold was additionally introduced. This was based on the observation that evaluation results are highly influenced by the chosen threshold values. In this study, results were analyzed using (1) a general threshold and (2) an optimal threshold that yielded the best performance for each sensor.

The general threshold was derived by averaging the anomaly scores at actual anomaly points from a subdataset of the MVTecAD image dataset [38]. In contrast, the optimal threshold was determined by adjusting various threshold values and selecting the one that produced the best performance.

Table 6 presents the results of uni-variate time series anomaly detection experiments conducted on the SWaT dataset using individual sensors. Since the ground truth used in the experiments includes attack information for all sensors, it is possible that an attack is marked in the ground truth, even if no attack occurred on the specific sensor being evaluated.

When applying the general threshold, the results showed superior performance compared to the baseline. Additionally, when optimal thresholds were set for each sensor, improvements were observed in the Recall and F1 score for most sensors, indicating enhanced detection performance for abnormal data. This suggests that the general threshold was stricter than the optimal threshold, as it typically had a higher value, leading to more stringent anomaly detection criteria.

For sensors such as DPIT-301 and AIT-504, the results were identical or very similar. This was because the values of the optimal threshold and the general threshold for these sensors were closely aligned. Overall, detection performance was particularly strong when the data exhibited general patterns and when the train and test datasets had similar characteristics. This is because the process of segmenting normal patterns and generating images facilitates the learning of normal data, thereby enabling more effective detection of anomalies that deviate from these patterns.

However, differences in the data patterns of individual sensors led to varying levels of normal pattern formation, resulting in some variation in anomaly detection performance. Nevertheless, the overall performance was consistently strong when optimal thresholds were applied to each sensor.

Table 7 presents the results of uni-variate time series anomaly detection experiments conducted on the UCR and NAB datasets. The experimental results for the UCR dataset demonstrated the best performance compared to existing models, which can be attributed to the characteristics of the dataset. The time series data in the UCR dataset consist of similar normal patterns, and the length of each pattern is neither too short nor too long. This allowed the data to be fully utilized without requiring additional preprocessing steps such as down-sampling. Furthermore, the differences in normal patterns between the training and testing data were minimal, making it relatively easier to detect anomalies when they appeared in the test set. These factors contributed to the superior performance observed on the UCR dataset compared to other datasets.

On the other hand, the results for the NAB dataset showed a Recall of 1.0, successfully detecting all anomalies, but the Precision and F1 score were relatively low. According to the dataset statistics reported for the baseline model TranAD [14], the anomaly ratio was 0.92%. However, in the actual data used for our experiments, the anomaly ratio was only 0.07%, with just 3 out of 4033 sequences labeled as anomalies. While the data surrounding the labeled anomalies exhibited significant deviations from the normal range, the Ground Truth designated only three specific points as anomalies.

Our system detected not only the labeled anomalies, but also the surrounding points affected by those anomalies, resulting in a relatively high number of False Positives and, consequently, lower Precision. However, since the system did not misclassify normal points outside the vicinity of anomalies as anomalies, the overall anomaly detection performance can still be regarded as robust.

Additionally, the experimental result of TSI-GAN [5], which employs a mechanism similar to ours by converting time series data into images and applying image anomaly detection techniques, achieved an F1 score of 0.846 on the InternalBleeding dataset. While there is a difference in the amount of data used, our model achieved a higher average F1 score of 0.939 based on experiments conducted on additional subdatasets.

Table 8 presents the results of multivariate time series anomaly detection experiments conducted on the SWaT dataset. The results of T2IAE are based on using the same GAF method as our system. In this study, anomaly detection was performed on a selected set of representative sensors from the SWaT dataset, and the results were aggregated to derive the final performance, which was then compared with baseline models. The experiments analyzed the results using a general threshold applied uniformly across all sensors and optimal thresholds tailored for each sensor.

**Figure 10:**
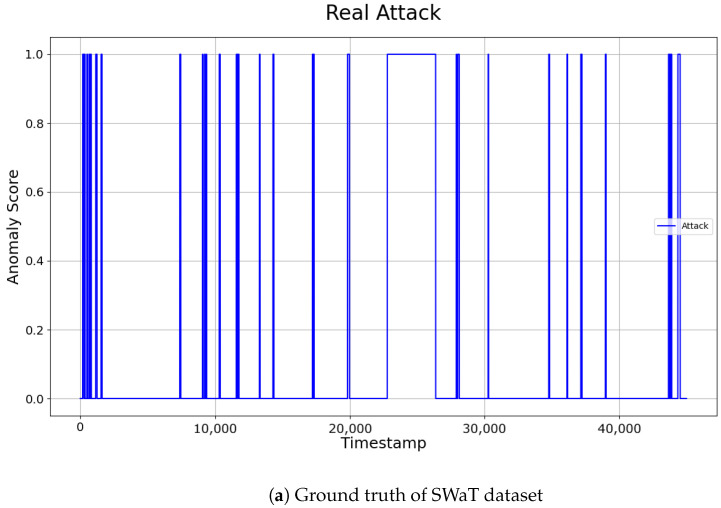

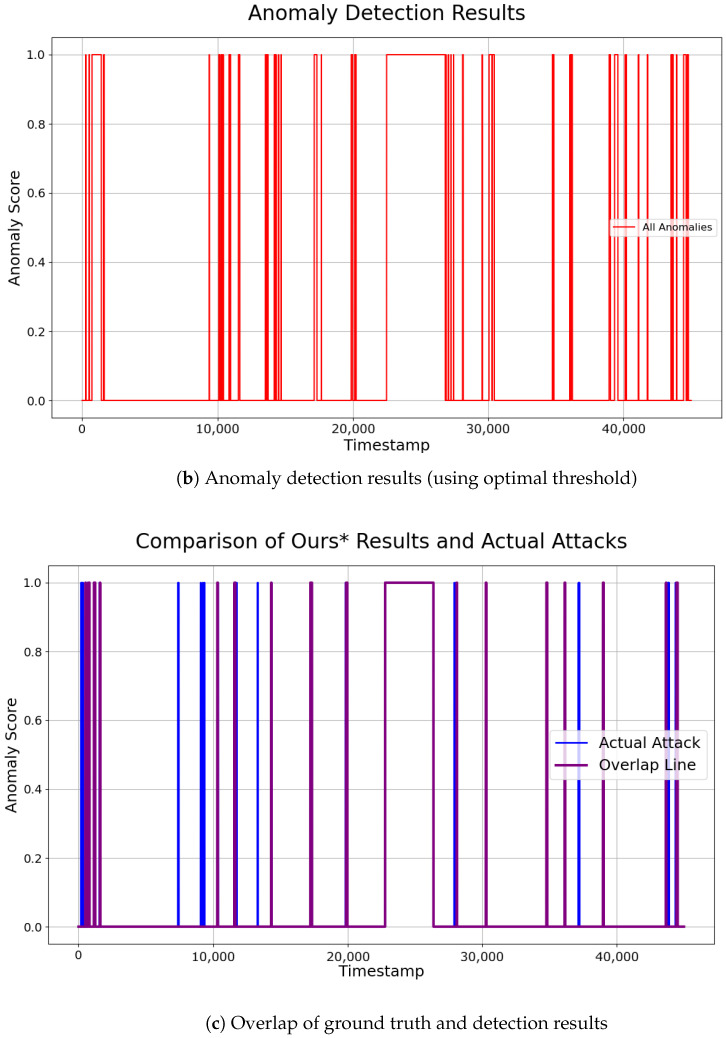
Visualization of ground truth and detection results on SWaT dataset.

The comparison of the two approaches showed that applying optimized thresholds for individual sensors reduced False Positives, and enabled the detection of more anomalies. The final performance of the SWaT dataset was evaluated by integrating the anomaly points detected across the selected sensors. The anomaly detection results can be intuitively observed in Figure 10. Our system demonstrated relatively low precision, but consistently high recall. This indicates that the model identified a larger number of points as anomalies, effectively maintaining a low False Positive Rate (FPR). The ability to identify anomalies without missing them, even at the cost of misclassifying some normal data as abnormal (False Positives), is often considered more valuable in certain domains. Therefore, the results of this study can be considered significant, especially in fields where rapid and reliable anomaly detection is crucial.

For this experiment, the analysis was conducted using representative sensors from similar sensor groups where attacks occurred, rather than averaging performance across all sensors in the SWaT dataset. Future research could consider the correlations between sensors and expand anomaly detection to a larger set of sensors. By aggregating results across more sensors, the system’s anomaly detection performance is expected to be further enhanced.

## 6. Discussion and Conclusions

This study consists of four independent modules: preprocessing, encoding time series, image anomaly detection, and anomaly detection in time series. The system flexibly captures normal patterns and considers temporal correlations, thereby generalizing them into image representations of normal patterns that are robust to minor fluctuations. This enables the construction of generalized normal patterns, effectively distinguishing abnormal data and enhancing the performance of unsupervised time series anomaly detection. Additionally, the system leverages anomaly maps derived from the image anomaly detection model to propose an efficient and effective detection algorithm. As a result, it achieves higher recall compared to previous studies. However, the quality of normal pattern formation significantly impacts detection performance; when the relationships between variables or subsequences are highly interdependent, it can be challenging to form normal patterns, which can negatively affect detection performance. These limitations can be addressed by adopting more advanced techniques tailored to the characteristics of the data.

This study preprocesses time series data and converts them into images for anomaly detection, but it cannot consider the relationships between transformed images. This limitation may result in performance degradation when processing time series data with non-cyclic or dynamic patterns, as it fails to capture inter-sequence correlations. To address this, our future research will work on developing methods to consider relationships between transformed images.

Furthermore, reflecting interdependency between variables for multivariate time series data could enable more advanced anomaly detection. To achieve this, we aim to improve image transformation techniques or incorporate additional methods such as convolution and attention mechanisms to enhance performance in multivariate time series anomaly detection.

In the image anomaly detection module, we utilized a knowledge-distillation-based model for efficient anomaly detection, but there are also studies based on various other techniques. We will continuously monitor trends in image anomaly detection to appropriately adopt and improve more efficient and high-performing models, thereby expanding the applicability of the system to diverse datasets and achieving competitive results.

In conclusion, the proposed method encodes time series data into images and applies them to image anomaly detection models, exploring the potential for cross-domain integration. It demonstrated strong performance with high recall, particularly on univariate time series datasets with cyclic patterns. Our system is expected to provide significant value in real-time applications requiring high anomaly detection rates, such as industrial control systems (ICS), IoT environments, healthcare, and financial domains. 

## Figures and Tables

**Table 1:** Dataset statistics.

Dataset	Train	Test	Anomaly Rate
SWaT [32]	47,520	44,991	12.2091%
UCR [33]	31,700	5900	1.8813%
NAB [34,35]	3800	4033	0.0007%

**Table 2:** Performance comparison between two segmentation methods for time series data. P: Precision, R: Recall, AUC: Area Under the ROC curve, F1: F1 score. The best scores are highlighted in bold. A higher value indicates a better result.

Data	Sensor	Regular				Cycle			
		P ↑	R ↑	F1 ↑	AUC ↑	P	R	F1	AUC
SWaT	LIT-101	**0.9603**	0.5949	0.7347	0.7958	0.9013	**0.6552**	**0.7588**	**0.8226**
	LIT-301	0.9069	0.6334	0.7458	0.8122	**0.9470**	**0.6858**	**0.7955**	**0.8402**
	AIT-202	0.1258	0.7542	0.2157	0.5127	**0.9207**	**0.6364**	**0.7526**	**0.8144**
	AIT-504	0.1184	**0.7883**	0.2059	0.4861	**0.9735**	0.5811	**0.7278**	**0.7895**
	MV-101	0.0824	0.0446	0.0579	0.4877	**0.8720**	**0.6537**	**0.7473**	**0.8202**
	MV-303	0.2161	0.0380	0.0647	0.5094	**0.9337**	**0.6714**	**0.7811**	**0.8324**
	DPIT-301	0.7397	**0.6787**	0.7079	**0.8227**	**0.9761**	0.6100	**0.7515**	0.8044
UCR		0.9412	**1.0000**	0.9697	**0.9994**	**0.9910**	0.9821	**0.9865**	0.9910

**Table 3:** Performance comparison between LGAF and GGAF. P: Precision, R: Recall, AUC: Area Under the ROC curve, F1: F1 score. The best scores are highlighted in bold.

Data	Sensor	LGAF				GGAF			
		P	R	F1	AUC	P	R	F1	AUC
SWaT	LIT-101	**0.9283**	0.6363	0.7550	0.8147	0.9013	**0.6552**	**0.7588**	**0.8226**
	LIT-301	**0.9470**	**0.6858**	**0.7955**	**0.8402**	0.9386	0.6734	0.7842	0.8336
	AIT-202	**0.9229**	0.6343	0.7518	0.8134	0.9207	**0.6364**	**0.7526**	**0.8144**
	AIT-504	**0.9735**	0.5811	**0.7278**	0.7895	0.9484	**0.5855**	0.7240	**0.7905**
	MV-101	0.8504	**0.6656**	0.7467	**0.8246**	**0.8720**	0.6537	**0.7473**	0.8202
	MV-303	**0.9337**	**0.6714**	**0.7811**	**0.8324**	0.9330	0.6714	0.7809	0.8323
	DPIT-301	0.9761	0.6100	0.7515	0.8044	**1.0000**	**0.6106**	**0.7582**	**0.8053**
UCR		**0.9910**	0.9821	**0.9865**	0.9910	0.9412	**1.0000**	0.9697	**0.9994**
NAB		**0.0182**	0.6667	**0.0354**	0.8199	0.0132	**1.0000**	0.0260	**0.9721**

**Table 4:** Performance comparison between RD and EAD. P: Precision, R: Recall, AUC: Area Under the ROC curve, F1: F1 score. The best scores are highlighted in bold.

Data	Sensor	RD				EAD			
		P	R	F1	AUC	P	R	F1	AUC
SWaT	LIT-101	**0.9310**	0.6308	0.7520	0.8121	0.9013	**0.6552**	**0.7588**	**0.8226**
	LIT-301	0.8981	**0.6881**	0.7792	0.8386	**0.9470**	0.6858	**0.7955**	**0.8402**
	AIT-202	**0.9279**	0.6304	0.7508	0.8118	0.9207	**0.6364**	**0.7526**	**0.8144**
	AIT-504	**0.9735**	0.5811	**0.7278**	0.7895	0.9369	**0.5922**	0.7257	**0.7933**
	MV-101	**0.8720**	0.6537	**0.7473**	**0.8202**	0.8627	**0.6545**	0.7443	0.8200
	MV-303	**0.9337**	0.6714	**0.7811**	0.8324	0.9134	**0.6758**	0.7768	**0.8334**
	DPIT-301	**0.9761**	0.6100	**0.7515**	0.8044	0.6434	**0.7042**	0.6724	**0.8249**
UCR		0.8279	0.9018	0.8632	0.9491	**0.9910**	**0.9821**	**0.9865**	**0.9910**
NAB		**0.0171**	**1.0000**	**0.0337**	**0.9787**	0.0132	1.0000	0.0260	0.9721

**Table 5:** Comparison of memory usage, train time, and anomaly map generation time between RD and EAD.

Methods	Train Time (s)	Test Time (s)	Memory Usage (MiB)
RD	1069	16	8978
EAD	2539	17	2030

**Table 6:** Performance comparison of anomaly detection methods on selected SWaT dataset sensors. Accu: Accuracy, P: Precision, R: Recall, F1: F1 score, FPR: Fall Positive Ratio. The best scores are highlighted in bold. A higher value indicates a better result. For FPR, a lower value indicates a better result.

Sensor	Methods	Accu ↑	P	R	F1	FPR ↓
LIT-101	CUSUM [39]	0.8663	0.3642	0.2686	0.0030	0.0739
	GAN-AD [40]	0.8763	0.5000	0.0175	0.0003	0.0932
	**Ours**	**0.9492**	**0.9310**	0.6308	0.7520	**0.0065**
	Ours *	0.9491	0.9013	**0.6552**	**0.7588**	0.0100
LIT-301	CUSUM	0.8150	0.1292	0.0902	0.0010	0.0843
	GAN-AD	0.8685	0.2222	0.0104	0.0002	**0.0052**
	**Ours**	0.9544	**0.9651**	0.6501	0.7769	**0.0033**
	Ours *	**0.9569**	0.9470	**0.6858**	**0.7955**	0.0053
AIT-202	CUSUM	0.5567	0.0924	0.2515	0.0013	0.3945
	GAN-AD	0.6022	0.0458	0.1710	0.0007	0.3548
	**Ours**	**0.9489**	**0.9279**	0.6304	0.7508	**0.0068**
	Ours *	0.9489	0.9207	**0.6364**	**0.7526**	0.0076
DPIT-301	CUSUM	0.8413	0.1846	0.1714	0.0017	0.0841
	GAN-AD	0.8440	0.2500	0.0267	0.0005	**0.0118**
	**Ours**	0.9199	0.6643	**0.6958**	0.6797	0.0489
	Ours *	**0.9203**	**0.6665**	0.6949	**0.6804**	0.0484
AIT-504	CUSUM	0.7097	0.0623	0.1438	0.0008	0.2301
	GAN-AD	0.8603	0.1474	0.1435	0.0014	0.1114
	**Ours**	0.9474	0.9624	0.5922	0.7332	0.0032
	Ours *	**0.9474**	**0.9624**	**0.5922**	**0.7332**	**0.0032**
MV-303	CUSUM	0.7155	0.0967	0.1918	0.0012	0.2201
	GAN-AD	0.8768	0.1754	0.0300	0.0005	0.0174
	**Ours**	0.9460	0.8720	0.6537	0.7473	0.0133
	Ours *	**0.9541**	**0.9337**	**0.6714**	**0.7811**	**0.0066**

Ours: general threshold; Ours *: optimal threshold.

**Table 7:** Performance comparison across UCR, NAB datasets. P: Precision, R: Recall, AUC: Area Under the ROC curve, F1: F1 score. The best scores are highlighted in bold.

Methods	UCR				NAB			
	P	R	F1	AUC	P	R	F1	AUC
MERLIN [41]	0.8013	0.7262	0.8414	0.7619	0.7542	0.8018	0.8984	0.7773
LSTM-NDT [42,43]	0.6400	0.6667	0.8322	0.6531	0.5231	0.8294	0.9781	0.6416
DAGMM [44]	0.7622	0.7292	0.8572	0.7453	0.5337	0.9718	0.9916	0.6890
OmniAnomaly [45]	0.8421	0.6667	0.8330	0.7442	0.8346	0.9999	0.9981	0.9098
MSCRED [46]	0.8522	0.6700	0.8401	0.7502	0.5441	0.9718	0.9920	0.6976
MAD-GAN [47]	0.8666	0.7012	0.8478	0.7752	0.8538	0.9891	0.9984	0.9165
USAD [48]	0.8421	0.6667	0.8330	0.7442	0.8952	1.0000	0.9989	0.9447
MTAD-GAT [49]	0.8421	0.7272	0.8221	0.7804	0.7812	0.9972	0.9978	0.8761
CAE-M [50]	0.7918	0.8019	0.8019	0.7968	0.6981	1.0000	0.9957	0.8222
GDN [15]	0.8129	0.7872	0.8542	0.7998	0.6894	0.9988	0.9959	0.8158
TranAD [14]	0.8889	**0.9892**	0.9541	0.9364	**0.9407**	1.0000	**0.9994**	0.9694
**Ours**	**0.9910**	0.9821	**0.9865**	**0.9910**	0.0171	**1.0000**	0.0337	**0.9787**

**Table 8:** Performance comparison across SWaT datasets. The results for SWaT are aggregated outcomes derived from a selection of representative sensors. Ours represents the results obtained with a general threshold, and SWaT* represents the results obtained with an optimal threshold. P: Precision, R: Recall, AUC: Area Under the ROC curve, F1: F1 score. The best scores are highlighted in bold.

Methods	SWaT			
	P	R	F1	AUC
MERLIN	0.6560	0.2547	0.6175	0.3669
LSTM-NDT	0.7778	0.5109	0.7140	0.6167
DAGMM	0.9933	0.6879	0.8436	0.8128
OmniAnomaly	0.9782	0.6957	0.8467	0.8131
MSCRED	**0.9992**	0.6770	0.8433	0.8072
MAD-GAN	0.9593	0.6957	0.8463	0.8065
USAD	0.9977	0.6879	0.8460	0.8143
MTAD-GAT	0.9718	0.6957	0.8464	0.8109
CAE-M	0.9697	0.6957	0.8464	0.8101
GDN	0.9697	0.6957	0.8462	0.8101
TranAD	0.9760	0.6997	**0.8491**	0.8151
T2IAE	0.9555	0.7611	0.8473	-
**Ours**	0.5673	0.7373	0.6412	0.8295
Ours *	0.6020	**0.7841**	0.6811	**0.8560**

Ours: general threshold; Ours *: optimal threshold. The baseline results are referenced from those provided in other papers and are presented accordingly. Metrics not officially reported are indicated with a dash (-).

## Data Availability

We used a total of three datasets in this study. The SWaT dataset is provided by iTrust, Centre for Research in Cyber Security, Singapore University of Technology and Design, upon request from the respective authors. If needed, the dataset can be requested through the following link: https://itrust.sutd.edu.sg/itrust-labs_datasets/dataset_info/ (accessed on 15 May 2023). The UCR dataset is a public dataset that is openly available at https://www.cs.ucr.edu/~eamonn/time_series_data/ (accessed on 17 May 2024). The NAB dataset is also a public dataset and can be accessed at https://github.com/numenta/NAB (accessed on 17 May 2024).

## Abbreviations

The following abbreviations are used in this manuscript:

GAF	Gramian Angular Field
LGAF	Local Gramian Angular Field
GGAF	Global Gramian Angular Field
KD	Knowledge Distillation
RD	Reverse Distillation
EAD	EfficientAD
PDN	Patch Description Network
SPADE	Semantic Pyramid Anomaly Detection
FAS	Final Anomaly Score
SWaT	Secure Water Treatment dataset
UCR	University of California, Riverside
NAB	Numenta Anomaly Benchmark
AWS	Amazon Web Services
MVTecAD	MVTec Anomaly Detection dataset
IoT	Internet of Things
